# Fluorescence Anion Chemosensor Array Based on Pyrenylboronic Acid

**DOI:** 10.3389/fchem.2020.00414

**Published:** 2020-05-28

**Authors:** Zhenbo Cao, Yang Cao, Riku Kubota, Yui Sasaki, Koichiro Asano, Xiaojun Lyu, Zhoujie Zhang, Qi Zhou, Xiaolei Zhao, Xu Xu, Si Wu, Tsuyoshi Minami, Yuanli Liu

**Affiliations:** ^1^Key Laboratory of New Processing Technology for Nonferrous Metal & Materials, Guilin University of Technology, Ministry of Education, Guilin, China; ^2^Institute of Industrial Science, The University of Tokyo, Tokyo, Japan

**Keywords:** anion, chemosensor array, boronic acid, fluorescence, regression analysis

## Abstract

A novel fluorescence chemosensor array composed of pyrenylboronic acid-based probes for multi- anion detection has been developed. The pyrenylboronic acid derivatives showed fluorescence quenching or enhancement due to photoinduced electron transfer originating from anion binding. The recognition ability was assessed by fluorescence titrations and electrospray ionization mass spectrometry. Because the array is constructed with cross-reactive probes, the combination of differential binding affinities for anions (i.e., fluoride, acetate, oxalate, malonate, citrate, dihydrogen phosphate, and pyrophosphate) and pattern recognitions, such as linear discriminant analysis, offered a successful simultaneous anion detection with a classification rate of 100%. Furthermore, the chemosensor array allowed for quantitative prediction of oxalate, malonate, and citrate in mixtures using a support vector machine. Importantly, the array system employs low-cost and commercially available reagents as probes. Thus, this study could lead to the development of user-friendly and high-throughput methods to detect a variety of analytes in complicated systems.

## Introduction

During the past decades, great efforts have been devoted to anion detection, because anions are essential to life and play important roles in industrial, environmental, and chemical processes (Schmidtchen and Berger, 1997; Gunnlaugsson et al., 2005; Gale and Dehaen, 2010). Although the methods for anion detection, such as mass spectrometry, have already been established (Berthod et al., 2018), use of large equipment is a potential drawback for on-site analysis. Furthermore, the diverse chemical and physical properties of anions limit their easy detection (Gómez et al., 2005; Mahoney et al., 2005; Sessler et al., 2006; Li et al., 2012; Chang et al., 2014; Edwards et al., 2015). Of all the methods for anion detection, we believe that the most powerful technique is chemosensing due to its advantages such as high sensitivity and applicability for rapid and high-throughput detection. Hence, a significant number of chemosensors have been developed for anion recognition *via* various capturing mechanisms (Amendola et al., 2010; Cavallo et al., 2010; Xu et al., 2010; Nishimuta et al., 2013; You et al., 2015; Mako et al., 2019). Among them, boronic acid derivatives have been widely utilized based on their reversible nature of binding with anions (Reetz et al., 1991; Cooper et al., 1998; Yamaguchi et al., 2001; Kubo et al., 2003, 2006; Koskela et al., 2005; Liu et al., 2005; Galbraith and James, 2010; Rout et al., 2012; Minami et al., 2016). In particular, the Lewis acidity allows boron-based chemosensors to act as a receptor for Lewis basic anions such as fluoride and phosphate (Wade et al., 2010; Wu X. et al., 2017). However, to the best of our knowledge, general methods to distinguish the multiple anions simultaneously using simple boronic acid-based chemosensors have not been established owing to the complex interference among analytes.

**Figure 1:**
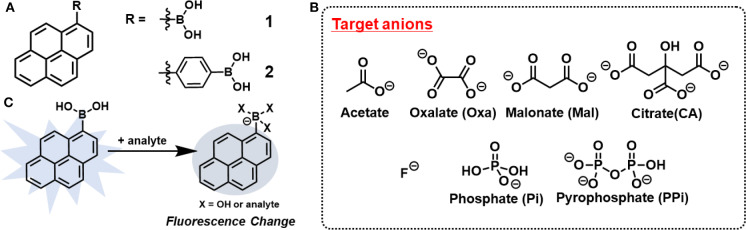
Chemical structures of **(A)** probes and **(B)** target anions in this study. **(C)** Schematic illustration for mechanism of anion recognition by **1**.

Chemosensor arrays constructed by multiple sensing units offer multi-target detection by utilizing crosstalk between sensors and analytes. The cross-reactivity in the array system provides a lot of advantages for practical applications such as reduction of cost and time of analysis. Therefore, chemosenor arrays were actively considered as those with the best methods to solve the above-mentioned challenge (Lavigne and Anslyn, 2001; Liu and Bonizzoni, 2014; Li et al., 2019). For example, Yamaguchi et al. synthesized a series of Zn(II)-dipicolylamine complexes covalently linked to anthracene for the detection of phosphate anions (Yamaguchi et al., 2005). Although these chemosensors were well designed according to the target analytes, their complicated structures may limit the practical applications of chemosensor arrays. Thus, it is of great importance and interest to develop chemosensor arrays using only simple materials. We previously reported indicator displacement assays utilizing only commercially available compounds, which realized the efficient discrimination of metal ions (Sasaki et al., 2017), saccharides (Sasaki et al., 2019a,b) and oxyanions (Hamedpour et al., 2019). Herein, we have employed a new combination of commercially available reagents to construct the chemosensor array in which the components are more simplified (Figure 1). Pyrenylboronic acid derivatives [1-Pyrenylboronic acid (**1**) and 4-(1-pyrenyl)phenylboronic acid (**2**)] were employed as probes because of their very simple chemical structures for fluorescence response (Wu D. et al., 2017). The simultaneous discrimination of seven anions was achieved by monitoring the fluorescence changes due to photoinduced electron transfer (PeT) (de Silva et al., 1997; Duke et al., 2010) originating from anion recognition. In addition, a quantitative analysis resulted in successful prediction of the concentrations of oxyanions (oxalate, citrate, and malonate) in the mixture.

## Materials and Methods

### Materials

The materials were purchased from commercial suppliers and used without further purification. The probes **1**, **2**, citric acid, and aqueous solution of tetra-*n*-butylammonium hydroxide (40% w/w) were purchased from Tokyo Chemical Industry Co. Ltd (Tokyo, Japan). Tetra-*n*-butylammonium salts of fluoride (F^−^), chloride (Cl^−^), bromide (Br^−^), iodide (I^−^), acetate, dihydrogen phosphate (Pi), and pyrophosphate (PPi) were purchased from Sigma-Aldrich (St. Louis, MO, USA). Oxalic acid and malonic acid were purchased from FUJIFILM WAKO Chemicals Corp (Tokyo, Japan). The general procedure for the synthesis of tetra-*n-*butylammonium salts of oxalate, citrate and malonate was as follows: 5 mL of an aqueous solution of tetra-*n-*butylammonium hydroxide (40% w/w) was mixed with oxalic acid, citric acid or malonic acid in a 20 mL glass bottle. The pH value of the solution was neutralized by adding the acids, followed by vacuum drying at 50**°**C.

### Measurements

Fluorescence spectra were recorded on a HITACHI F-7100 spectrophotometer. Fluorescence titrations with anions were performed in DMSO, and the titration isotherms were obtained from changes in the fluorescence maxima (378 and 401 nm for **1** and **2**, respectively). The fluorescence titration was carried out under ambient conditions at 25**°**C. The binding constants were determined by fitting the fluorescence titration curves according to previous studies (Hargrove et al., 2010). Mass spectrometry was performed using a SHIMADZU LCMS-2020 spectrometer.

The array experiment for qualitative and quantitative analyses was performed by a Biotek SYNERGY H4 microplate reader. The details of the processes are as follows: the analyte was added to the probe solution at a certain concentration, and then the resulting mixing solution was shaken and allowed to stand for 30 min. After this period, the solution was transferred to the 96-well microplates and fluorescence spectra were recorded from 350 to 550 nm. The fluorescence measurements were repeated 24 times. A class of 20 repetitions with a variability coefficient lower than 9% was extracted by the Student's *t*-test. Then, the qualitative and semi-quantitative analyses were carried out using a linear discriminant analysis (LDA) without any further pretreatment (Brereton, 2007; Anzenbacher et al., 2010). A support vector machine with a principal component analysis preprocessing (PCs = 3) was used for the analysis of quantitative assay (Hamel, 2009).

## Results and Discussion

**Figure 2:**
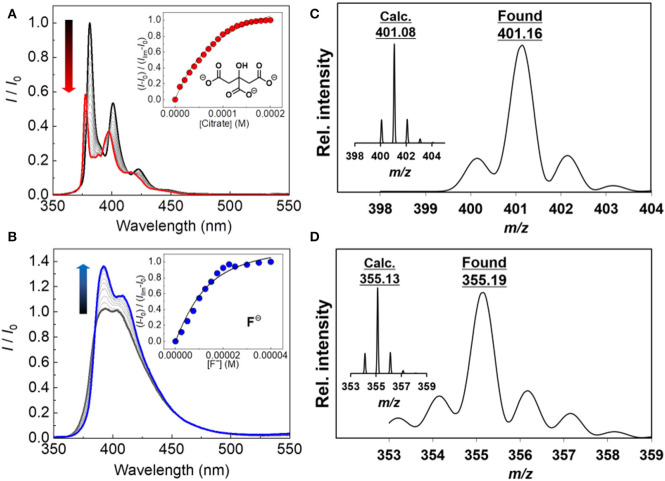
Fluorescence spectra of **(A) 1** in DMSO upon addition of citrate at 25**°**C (0–200 μM) and **(B) 2** in DMSO upon addition of fluoride at 25**°**C (0–40 μM). [**1**] = [**2**] =10 μM, λ_ex_ = 340 nm for **1** and **2**. ESI-MS spectrum (negative) of **(C)** the **1**–citrate complex (inset: calculated isotope pattern for C_22_H_14_${\text{BO}}_{7}^{-}$), and **(D)** the **2**–fluoride complex (inset: calculated isotope pattern for C_23_H_17_${\text{BFO}}_{2}^{-}$).

To examine the binding of the boronic acid-based probes (**1** and **2**) with various anions, the fluorescence titrations were carried out at 25**°**C. The spectra are shown in Figures 2A,B and Supplementary Materials. The fluorescence spectra of probe **2** were broader than those of probe **1**. 1-Aryl substituted pyrene derivatives reported a loss of vibrational structure in UV-vis absorption and fluorescence properties (Weigel et al., 2003), meaning that separation of S_2_ ← S_0_ and S1 ← S_0_ excitations is almost impossible. All orbitals including the above excitations possess nonzero contributions at the 1-position, resulting in the significant influence on both transitions (Crawford et al., 2011). The probe **1** exhibited fluorescence increase and decrease depending on the types of analytes tested. The fluorescence increase was observed in the presence of acetate, Pi, and PPi, while the other types of anions (oxalate, citrate, malonate, and fluoride) resulted in fluorescence decrease. In case of probe **2**, the addition of any type of anions increased the fluorescence intensity. The fluorescence ON-OFF and OFF-ON features are presumably due to the PeT between fluorophore and the boron atom (Yoon and Czarnik, 1992; Sandanayake et al., 1995; Sun et al., 2019), which may be affected by the difference of the electronic delocalization. To address the mechanism of the observed PeT phenomenon, we measured UV-Vis spectra of the probes in the presence or absence of citrate as an example of the target anions. The probe **2** showed no UV-Vis spectral change, which supports the PeT mechanism (de Silva et al., 1997). On the other hand, slight UV-Vis and fluorescence spectral shifts were observed for the probe **1**, indicating that a weak intramolecular charge transfer would co-occur with the PeT (see Supplementary Material) (Hagopian and Singer, 1985; de Silva et al., 1997; Schneider et al., 2001; Oesch and Luedtke, 2015; Sasaki et al., 2016). Among the targets, the highest response to anions was citrate. Probes **1** and **2** showed 76% of an on-off response (i.e., *I*_sat._/*I*_0_) and 162% of an off-on response, respectively. On the other hand, the probes showed very weak or no response to fructose which can potentially interfere the boronic acid-based anion detection (see Supplementary Material). In addition, the formation of the boronate species was also proven by electrospray ionization mass spectrometry (ESI-MS) (Figures 2C,D and Supplementary Material).

The stoichiometries for binding (i.e., anion-to-probe ratio) were estimated to be 1:1 except for Pi and PPi. From the fluorescence titration isotherms, the binding constants (*K*s) of the probes for anions were determined (Table 1). It is worth noting that the probes displayed different affinities for the anions, which resulted in a unique response of the probes to various guests. It is well known that the cross-reactivity between the probe and analyte is crucial for the classification rate of an array. Therefore, we envisioned that the fluorescent properties of the probe **1** and **2** contribute to the development of a new chemosensor array to discriminate multiple anions qualitatively and quantitatively.

**Table 1:** Binding constants (*K*, M^−1^)^a^ obtained from fluorescence titration.

**Anion^**b**^**	**Oxalate**	**Citrate**	**Malonate**	**Acetate**	**Pi**		**PPi**	***F*^**−**^**
					***K*_**11**_**	***K*_**21**_**		
1	(7.1 ± 0.5) × 10^4^	(1.1 ± 0.1) × 10^4^	(4.2 ± 0.8) × 10^3^	N.D.^c^	(1.6 ± 0.1) × 10^5^	(3.0 ± 0.1) × 10^4^	N.D.^c^	>10^6^
2	(1.1 ± 0.1) × 10^5^	N.D.^c^	(8.2 ± 1.0) × 10^4^	(1.3 ± 0.2) × 10^5^	<10^3^	> 10^8^	N.D.^c^	>10^6^

a *Binding constants were calculated using the change in fluorescence titrations at 25**°**C*.

b *Small response was observed for the addition of Cl^−^, Br^−^, and I^−^. Thus, the K values could not be calculated*.

c *The K value could not be calculated due to sigmoidal responses*.

**Figure 3:**
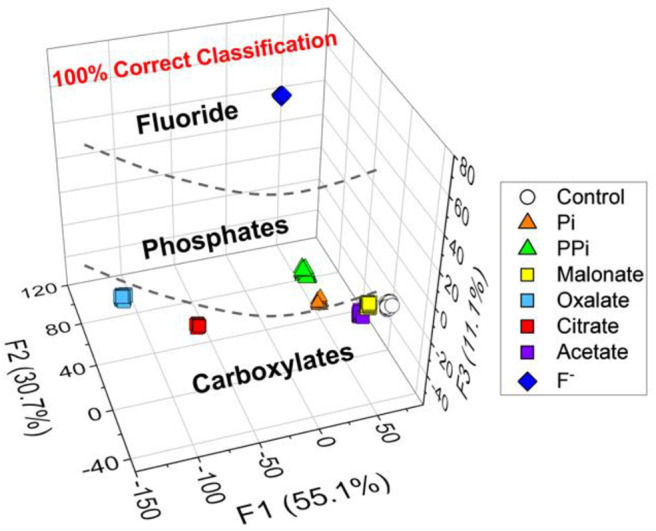
LDA plots for the response of 8 types of clusters (including a control sample). [Anion] = 200 μM. Twenty.repetitions were measured for each and the cross-validation routine shows 100% successful classification. The data were obtained by using the probes **1** and **2**.

Among the pattern recognition algorithms, a linear discriminant analysis (LDA) (Brereton, 2007) is one of the suitable supervised methods to test our hypothesis. We employed the LDA to reduce the dimensionality and to realize multivariate data classification. Additionally, the leave-one-out cross-validation protocol (namely the Jackknife method) was employed to evaluate the level of correct classification within the clusters (see Supplementary Material). To ensure the accuracy, the trials were repeated 24 times for each set of analytes, and then four outliers were eliminated by the Student's *t*-test. As shown in Figure 3, seven types of analytes and controls (160 data points) were clearly discriminated with a successful rate of 100%. This result proves our expectation that the new chemosensor array constructed of probe **1** and **2** possesses excellent capability for multiple anions detection.

**Figure 4:**
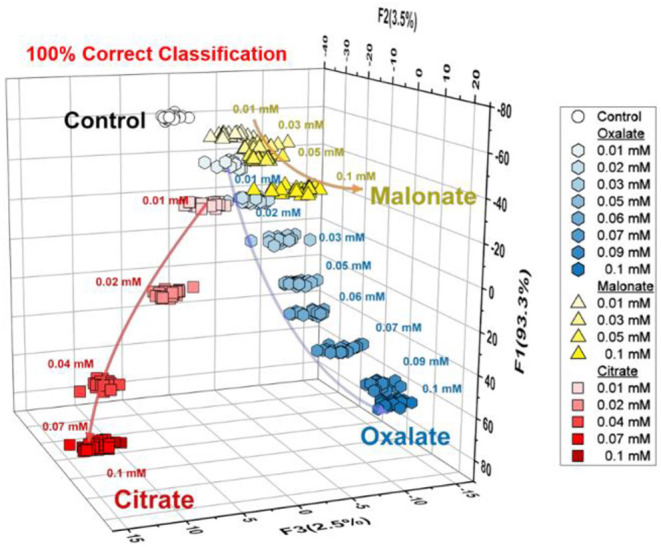
Semi-quantitative LDA plot for three types of anions (citrate, oxalate and malonate) at a concentration range of 0–100 μM. For each concentration, the trial was repeated 24 times. The data were obtained by using the probes **1** and **2**.

Taking advantage of LDA, the semi-quantitative assay was carried out to evaluate the capability of the array system for quantitative anion recognition. The analysis of the oxyanions (oxalate, citrate, and malonate) is shown in Figure 4. As can be seen, the LDA plots were clearly dependent not only on the concentration of anions but also the type, which is completely consistent with the results of the fluorescence titrations. This result encouraged us to employ the array for regression analysis of the anions in mixtures.

**Figure 5:**
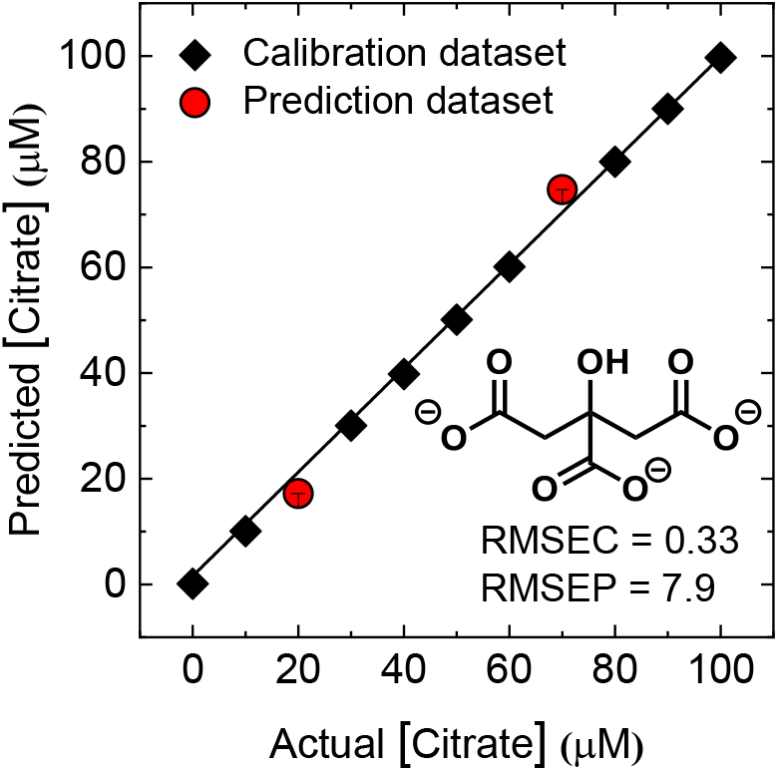
The results of the regression analysis for quantitative analysis of citrate in mixtures of oxyanions (oxalate, citrate, and malonate). The data were obtained by using the probes **1** and **2**. The detailed concentration conditions are summarized in Supplementary Material. Plots of actual vs. predicted concentrations show high accuracy of prediction for multiple concentrations of each analyte. The values of root-mean-square error (RMSE) of calibration (C) and prediction (P) prove the high quality of the model and prediction.

Figure 5 shows the results of the quantitative array for a mixture of the same concentrations of oxalate, citrate, and malonate. For the regression analysis, a support vector machine (SVM) algorithm, which is suitable for complex nonlinear responses, was employed. Here, we used nine analyte concentrations for model construction and two different analyte concentrations for prediction. Consequently, the unknown concentrations were successfully predicted by the constructed model with excellent root-mean-square errors (RMSE values). This indicated that the developed array can predate the concentration of oxalate, citrate, and malonate even in mixtures.

## Conclusion

In summary, a pyrenylboronic acid-based chemosensor array was constructed through a combination of simple commercially available reagents. The proposed array exhibited an excellent capability for discriminating between multiple anions in a qualitative as well as quantitative manner. In the qualitative analysis, seven types of anions were successfully discriminated by employing LDA with 100% correct classification. Moreover, the semi-quantitative assay for oxalate, citrate, and malonate showed species-dependent and concentration-dependent changes in the LDA plots. Thus, the regression analysis was performed and the concentration of individual components was successfully predicted in the mixture. To the best of our knowledge, fabrication of a boronic acid-based chemosensor array with the ability to discriminate between several anions has not previously been reported. Thus, the simple chemosensor array proposed in this study will provide a new platform for the application of chemosensors in the field of molecular recognition chemistry.

## Data Availability Statement

All datasets generated for this study are included in the article/Supplementary Material.

## Author Contributions

ZC, YC, RK, YS, KA, and XL performed the fluorescence and mass spectrometry experiments, and wrote the manuscript. ZZ, QZ, XZ, XX, and SW contributed equally in this work. TM and YL conceived the entire project.

## Conflict of Interest

The authors declare that the research was conducted in the absence of any commercial or financial relationships that could be construed as a potential conflict of interest.
