# Supplementary material for: Fluorescence Anion Chemosensor Array Based on Pyrenylboronic Acid
Source: Front Chem. 2020 May 28;8:414. doi: 10.3389/fchem.2020.00414 (PMC7272501; doi:10.3389/fchem.2020.00414)
Supplement: Supplementary file 1 [file Data_Sheet_1.PDF]

Supplementary Material

Fluorescence Anion Chemosensor Array Based on Pyrenylboronic Acid

Zhenbo Cao[1], Yang Cao[1], Riku Kubota[2], Yui Sasaki[2], Koichiro Asano[2], Xiaojun Lyu[2], Zhoujie Zhang[2], Qi Zhou[2], Xiaolei Zhao[1], Xu Xu[1], Si Wu[1], Tsuyoshi Minami[1,2*] and Yuanli Liu[1*]

[1]Key Laboratory of New Processing Technology for Nonferrous Metal & Materials, Guilin University of Technology, Ministry of Education, Guilin 541004, China

[2]Institute of Industrial Science, The University of Tokyo, 4-6-1 Komaba, Meguro-ku, Tokyo 153-8505, Japan

Contents

1. **^{1}H NMR, ^{13}C NMR, and ESI MS analyses of probes** **S2**
2. **Examples of UV-vis measurement for anion** **S2**
3. **Fluorescence measurements for anions** **S2**
4. **Selected ESI MS analysis** **S8**
5. **Linear discriminant analysis (LDA)** **S11**

1. ^{1}H NMR, ^{13}C NMR, and ESI MS analyses of probes

1: ^{1}H NMR (DMSO-d_6, 400 MHz, δ ppm): 8.71 (d, 1H, J = 9.2 Hz, Ar-H), 8.56 (s, 2H, OH), 8.30-8.24 (m, 4H, Ar-H), 8.21-8.17 (m, 3H, Ar-H), 8.07 (t, 1H, J = 7.6 Hz, Ar-H); ^{13}C NMR (DMSO-d_6, 100 MHz, δ ppm): 123.7, 124.0, 124.1, 125.0, 125.0, 126.0, 126.8, 127.5, 127.7, 128.5, 130.4, 130.8, 131.4, 131.7, 134.0 ; ESI-MS^{-} ($C_{18}H_{16}BO_3^{-}$): m/z 291.34.

2: ^{1}H NMR (DMSO-d_6, 400 MHz, δ ppm): 8.38 (d, 1H, J = 7.9 Hz, Ar-H), 8.34 (dd, 1H, J = 1.1 Hz, 7.6 Hz, Ar-H), 8.30 (d, 1H, J = 1.0 Hz, 7.8 Hz, Ar-H), 8.24 (m, 2H, Ar-H), 8.21 (s, 2H, OH), 8.20-8.17 (m, 1H, Ar-H), 8.13-8.09 (m, 2H, Ar-H), 8.05-8.02 (m, 3H, Ar-H), 7.62 (d, 2H, J = 8.2 Hz, Ar-H); ^{13}C NMR (DMSO-d_6, 100 MHz, δ ppm): 124.1, 124.2, 124.7, 125.0, 125.0, 125.4, 126.5, 127.4, 127.5, 127.6, 127.7, 127.8, 129.5, 130.2, 130.4, 131.0, 134.4, 137.2, 142.0; ESI-MS^{-} ($C_{24}H_{20}BO_3^{-}$): 367.36.

2. Examples of UV-vis measurement for anion

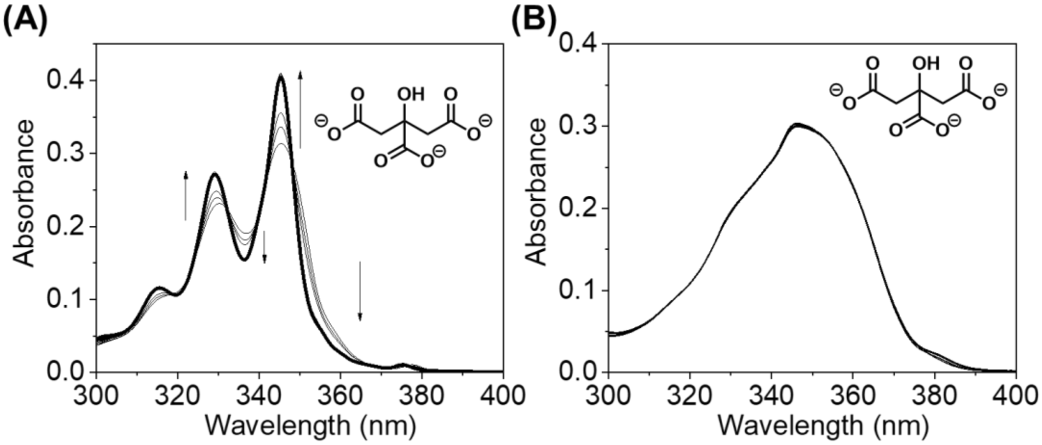

Supplementary Figure 1. UV-vis spectra of (A) **1** or (B) **2** in DMSO upon addition of citrate at 25 °C (0–1 mM). [**1**] = [**2**] =10 μM.

3. Fluorescence measurements for anions

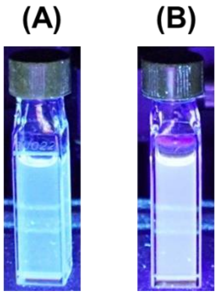

Supplementary Figure 2. Photographs of (A) **1** and (B) **1** with citrate in DMSO. [**1**] = 10 μM, [citrate] = 1 mM. The solutions were excited by a black light.

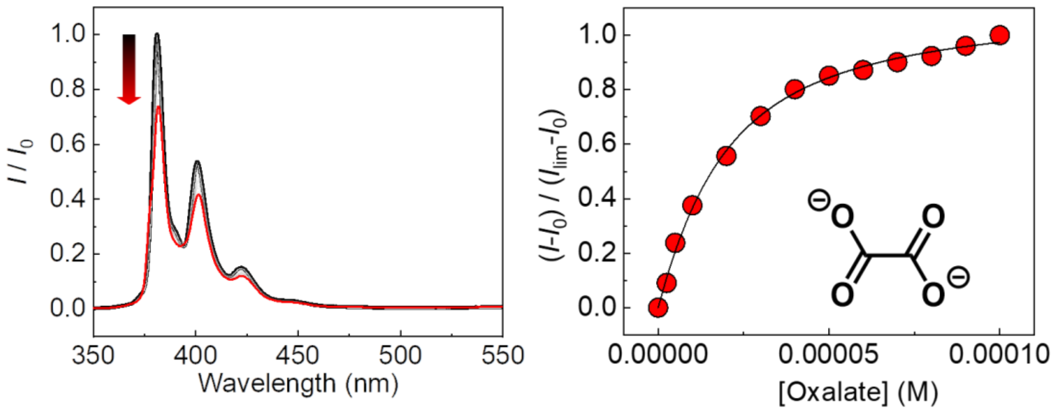

Supplementary Figure 3. Fluorescence spectra of **1** in DMSO upon addition of oxalate at 25 °C (0–100 μM). [**1**] = 10 μM, λ_{ex} = 340 nm.

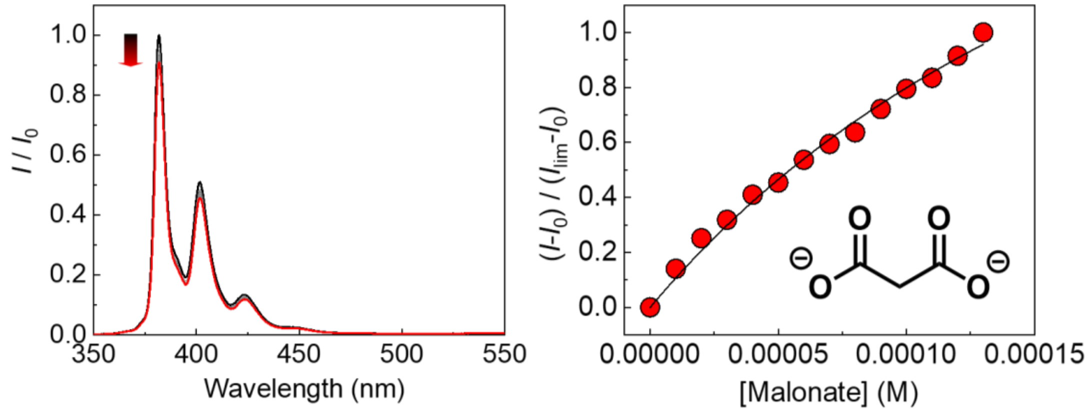

Supplementary Figure 4. Fluorescence spectra of **1** in DMSO upon addition of malonate at 25 °C (0 –130 μM). [**1**] = 10 μM, λ_{ex} = 340 nm.

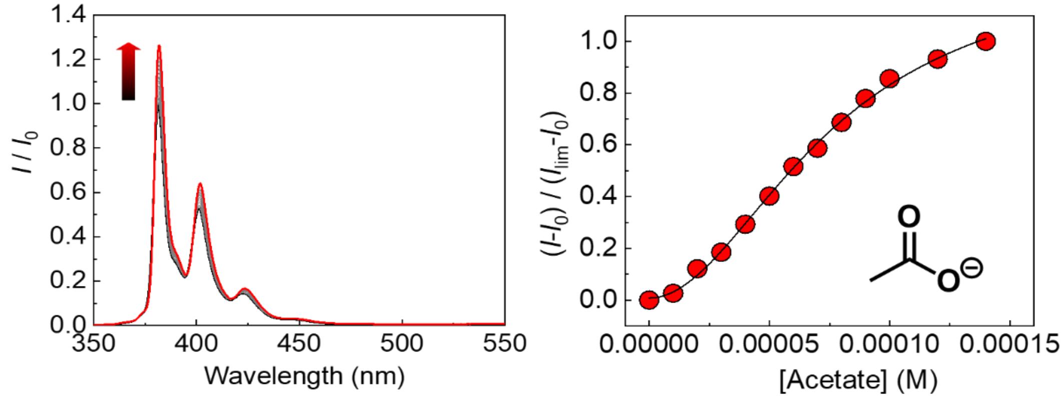

Supplementary Figure 5. Fluorescence spectra of **1** in DMSO upon addition of acetate at 25 °C (0 – 140 μM). [**1**] = 10 μM, λ_{ex} = 340 nm.

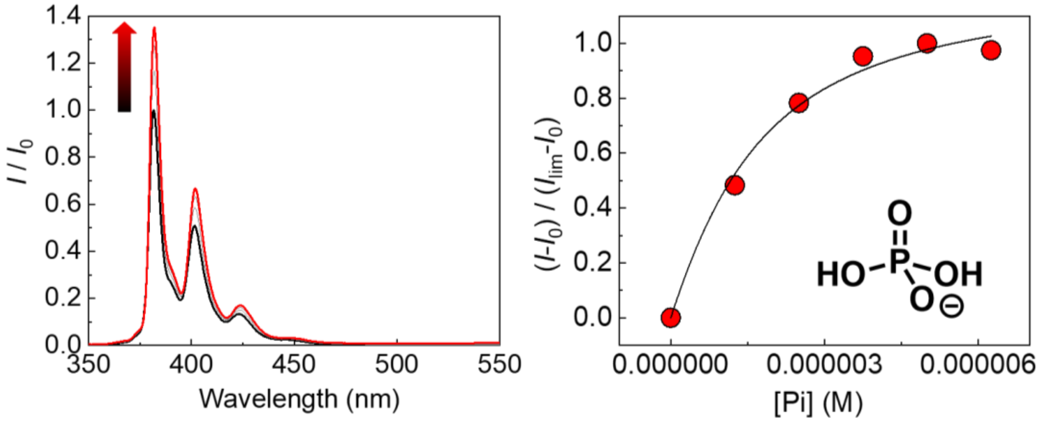

Supplementary Figure 6. Fluorescence spectra of **1** in DMSO upon addition of Pi at 25 °C (0 – 6 μM). [**1**] = 10 μM, λ_{ex} = 340 nm.

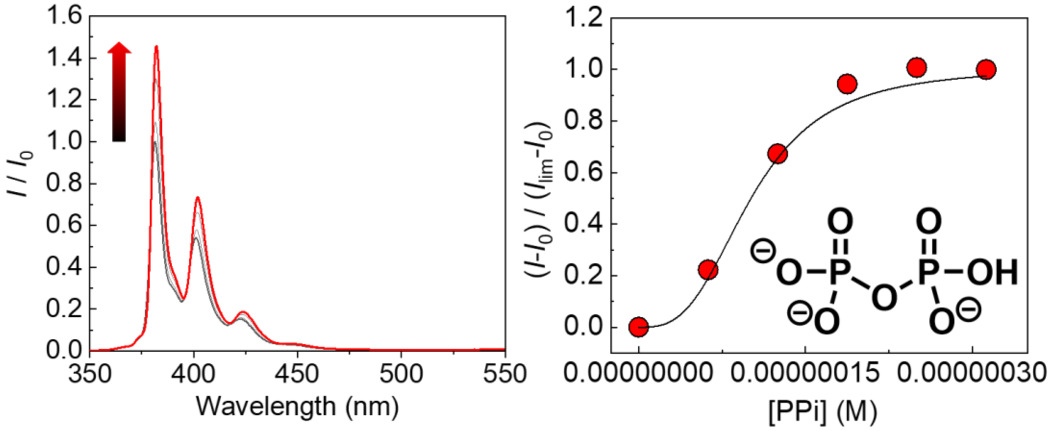

Supplementary Figure 7. Fluorescence spectra of **1** in DMSO upon addition of PPi at 25 °C (0 – 0.31 μM). [**1**] = 10 μM, λ_{ex} = 340 nm.

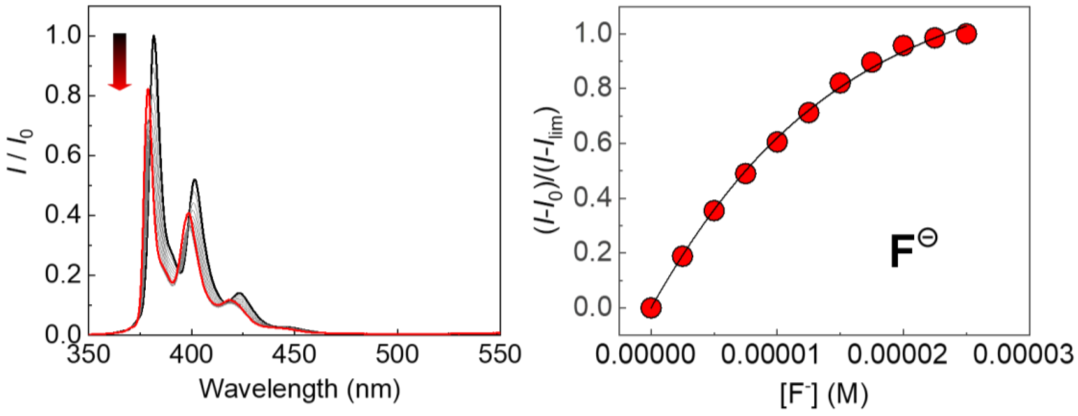

Supplementary Figure 8. Fluorescence spectra of **1** in DMSO upon addition of F^{-} at 25 °C (0 –25 μM). [**1**] = 10 μM, λ_{ex} = 340 nm.

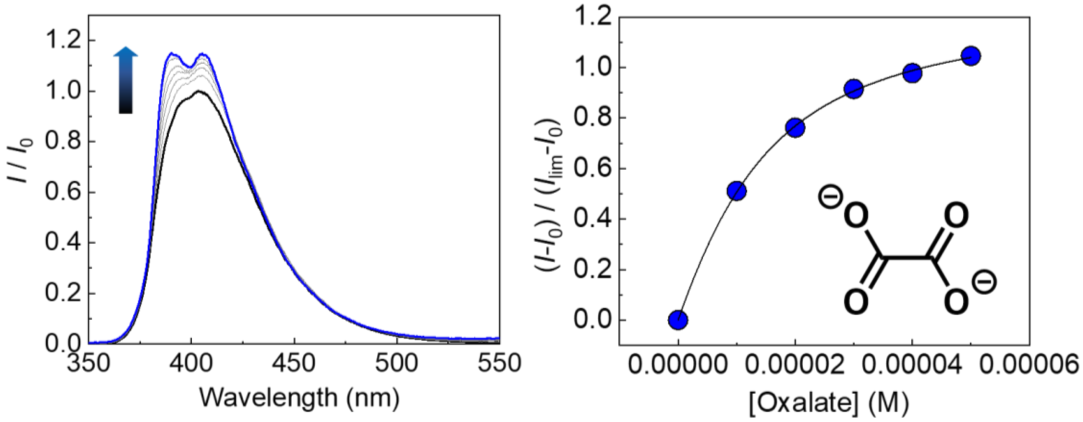

Supplementary Figure 9. Fluorescence spectra of **2** in DMSO upon addition of oxalate at 25 °C (0 – 50 μM). [**2**] = 10 μM, λ_{ex} = 340 nm.

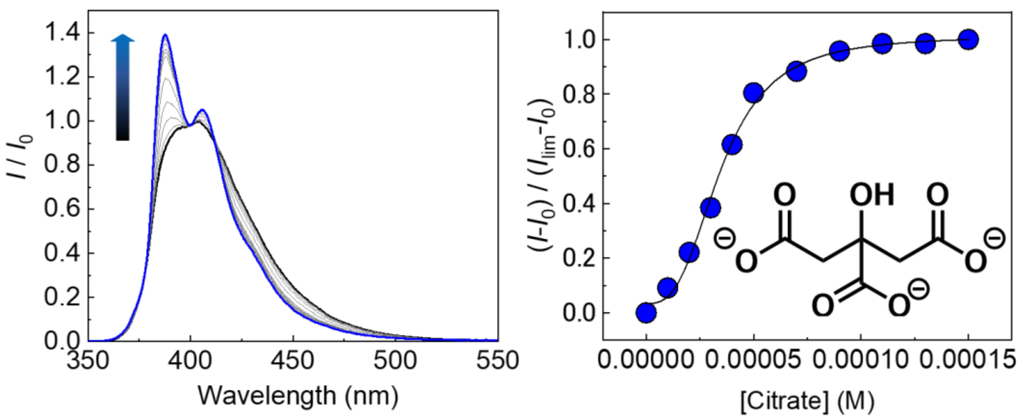

Supplementary Figure 10. Fluorescence spectra of **2** in DMSO upon addition of citrate at 25 °C (0 – 150 μM). [**2**] = 10 μM, λ_{ex} = 340nm.

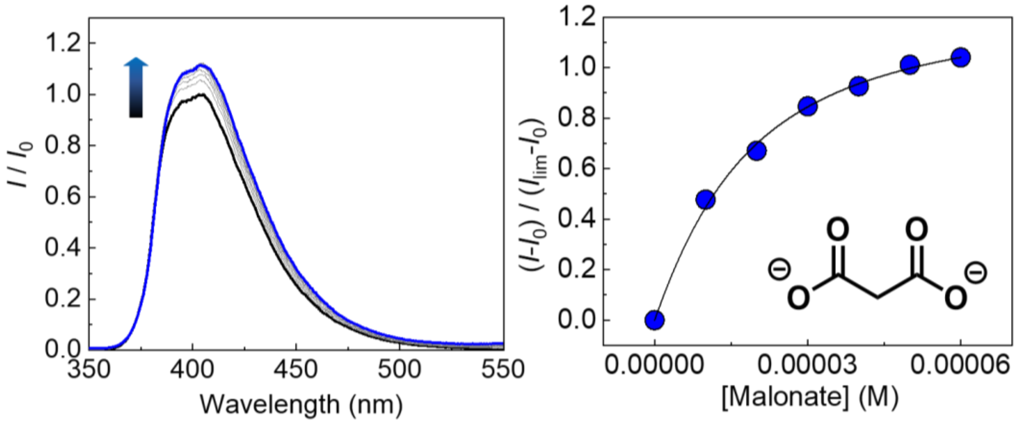

Supplementary Figure 11. Fluorescence spectra of **2** in DMSO upon addition of malonate (0 – 60 μM) at 25 °C. [**2**] = 10 μM, λ_{ex} = 340 nm.

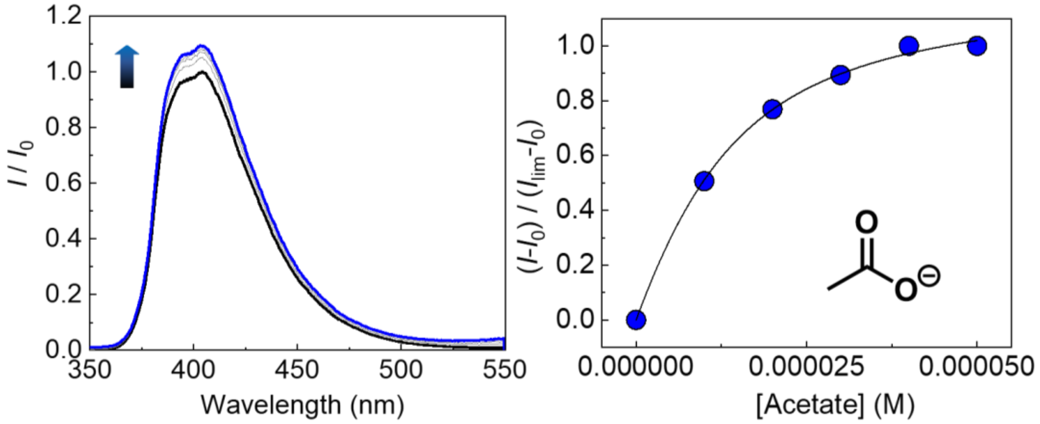

Supplementary Figure 12. Fluorescence spectra of **2** in DMSO upon addition of acetate at 25 °C (0 – 50 μM). [**2**] = 10 μM, λ_{ex} = 340 nm.

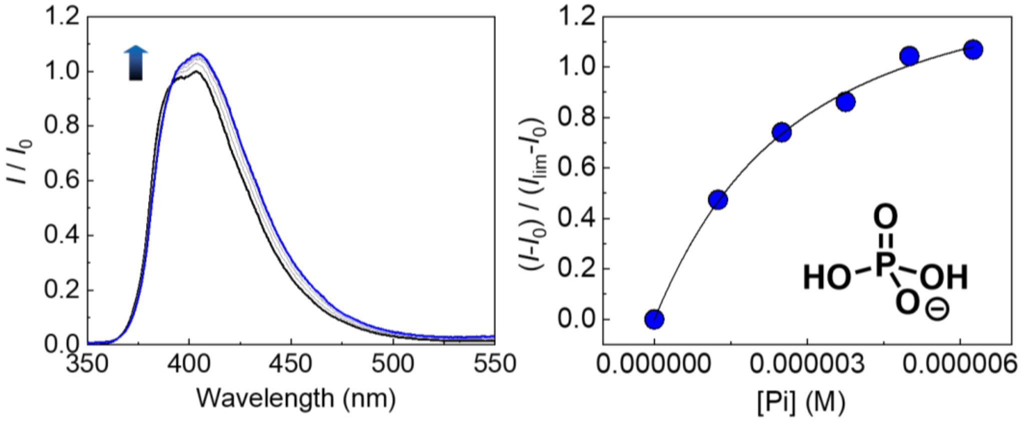

Supplementary Figure 13. Fluorescence spectra of **2** in DMSO upon addition of Pi at 25 °C (0 – 6 μM). [**2**] = 10 μM, λ_{ex} = 340nm.

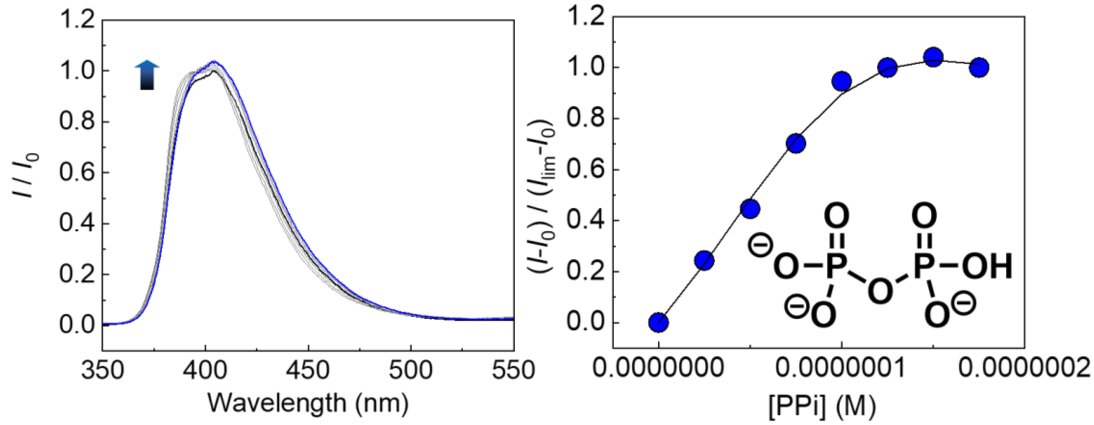

Supplementary Figure 14. Fluorescence spectra of **2** in DMSO upon addition of PPi at 25 °C (0 – 0.18 μM). [**2**] = 10 μM, λ_{ex} = 340 nm.

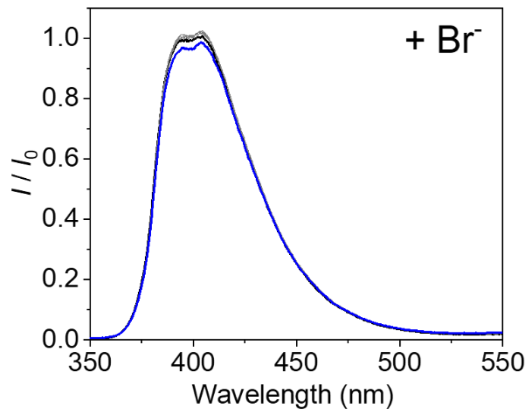

Supplementary Figure 15. Fluorescence spectra of **2** in DMSO upon addition of Br^- at 25 °C (0 – 300 μM). [**2**] = 10 μM, λ_{ex} = 340 nm.

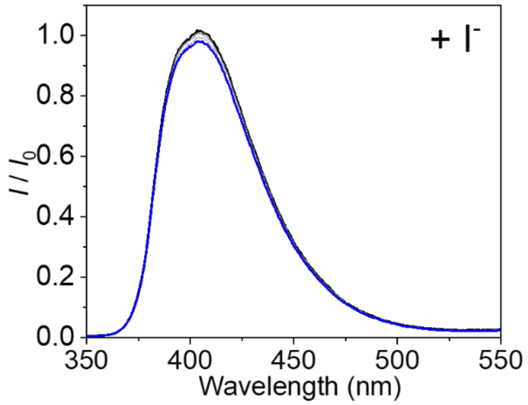

Supplementary Figure 16. Fluorescence spectra of **2** in DMSO upon addition of I^- at 25 °C (0 – 300 μM). [**2**] = 10 μM, λ_{ex} = 340 nm.

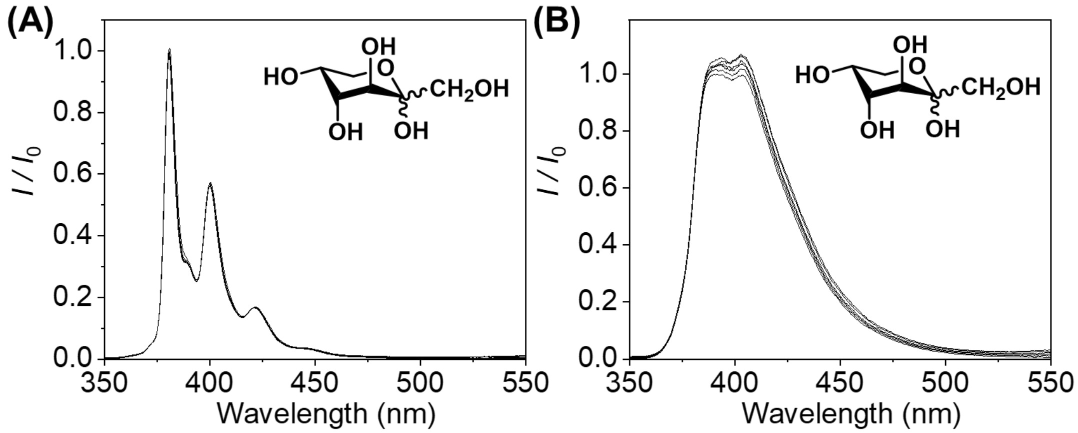

Supplementary Figure 17. Fluorescence spectra of (A) **1** and (B) **2** in DMSO upon addition of fructose at 25 °C (0 – 300 μM). [**1**] = [**2**] = 10 μM, λ_{ex} = 340 nm.

2. Selected ESI-MS analysis

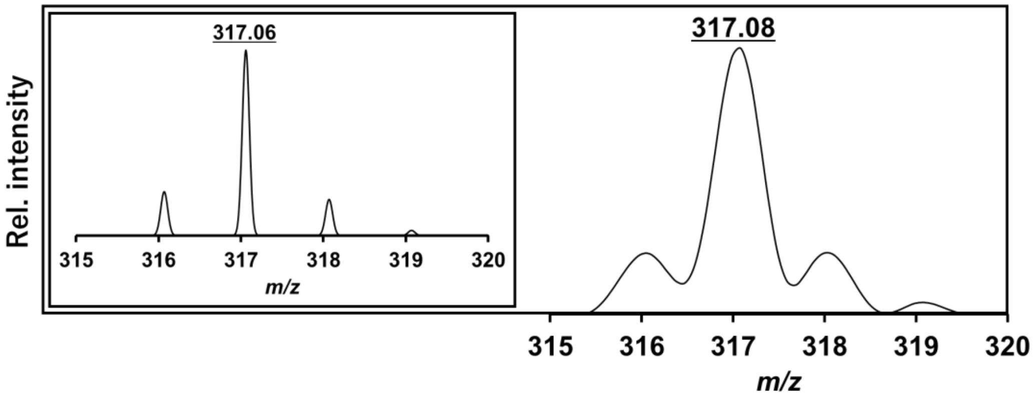

Supplementary Figure 18. ESI-MS spectrum (negative) of the **1**–oxalate complex. Inset: calculated isotopic pattern for $C_{18}H_{10}BO_5^-$.

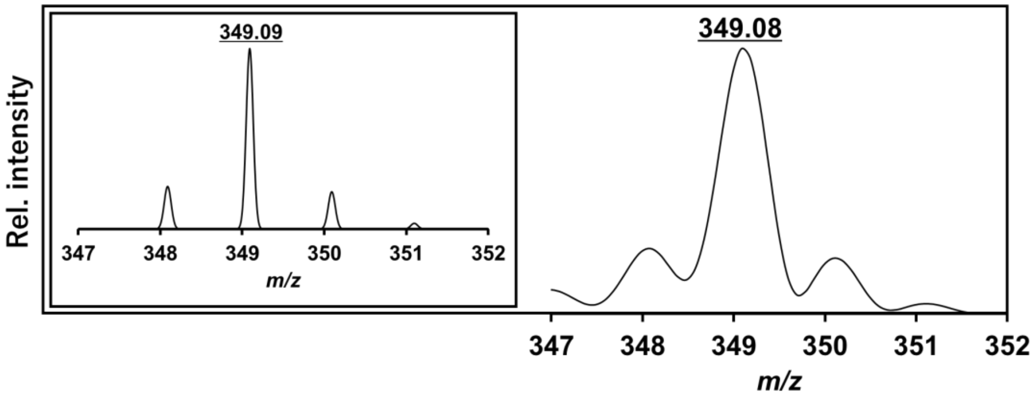

Supplementary Figure 19. ESI-MS spectrum (negative) of the **1**–malonate complex. Inset: calculated isotopic pattern for $C_{19}H_{14}BO_6^-$.

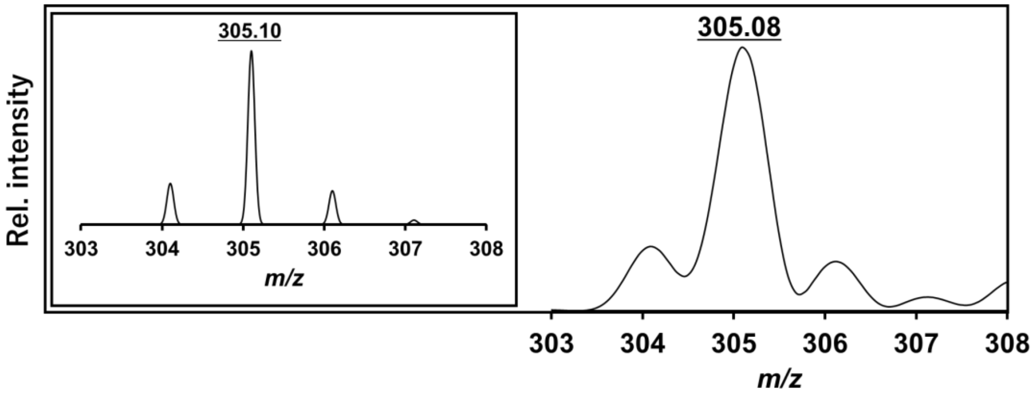

Supplementary Figure 20. ESI-MS spectrum (negative) of the **1**–acetate complex. Inset: calculated isotopic pattern for $C_{18}H_{14}BO_4^-$.

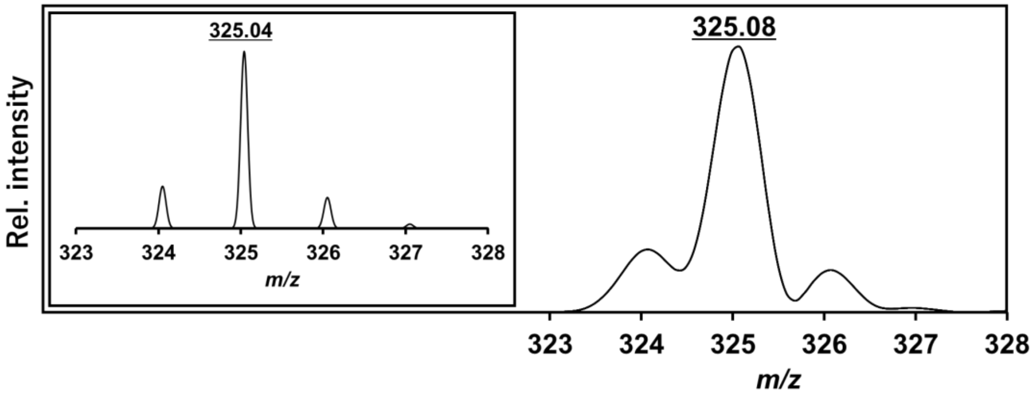

Supplementary Figure 21. ESI-MS spectrum (negative) of the **1**–Pi complex. Inset: calculated isotopic pattern for $C_{16}H_{11}BO_5P^-$.

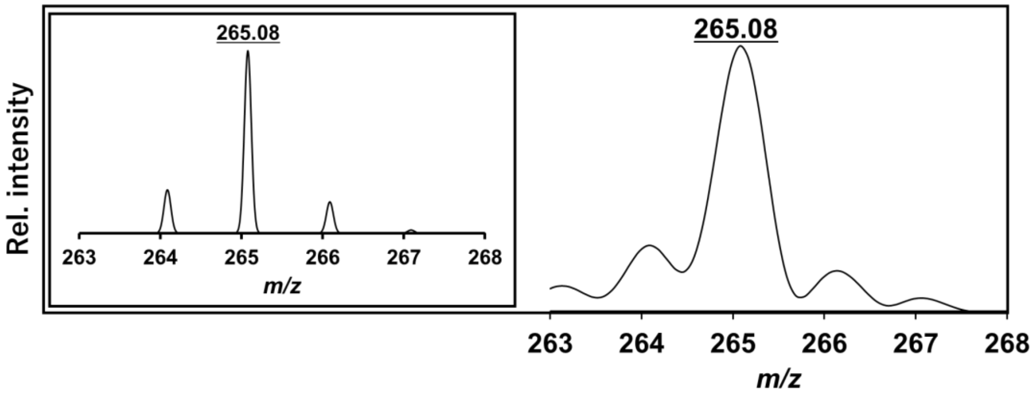

Supplementary Figure 22. ESI-MS spectrum (negative) of the **1**–fluoride complex. Inset: calculated isotopic pattern for $C_{16}H_{11}BFO_2^-$.

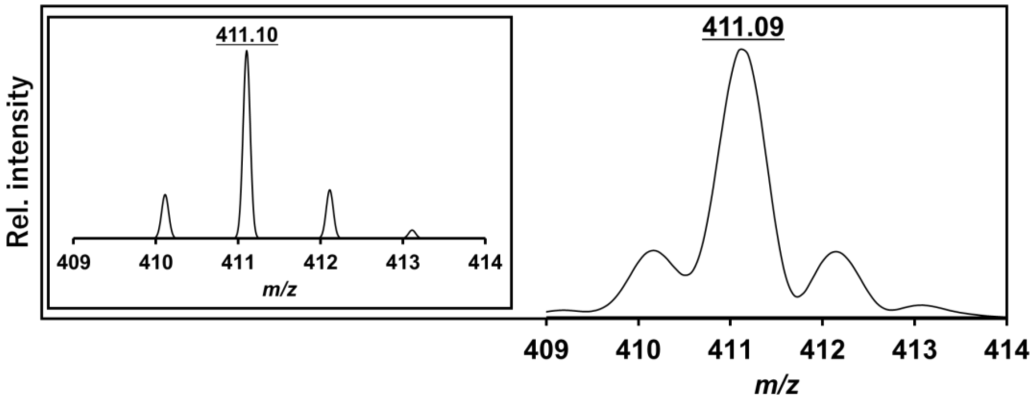

Supplementary Figure 23. ESI-MS spectrum (negative) of the **2**–oxalate complex. Inset: calculated isotopic pattern for $C_{24}H_{16}BO_6^-$.

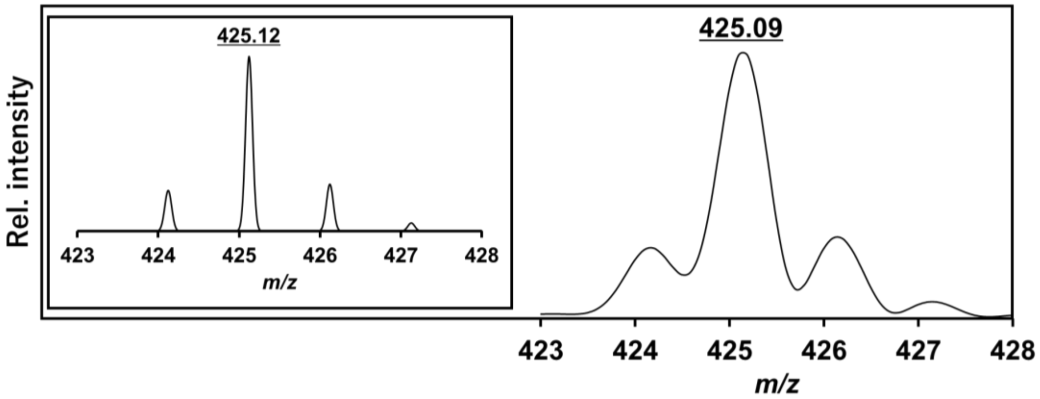

Supplementary Figure 24. ESI-MS spectrum (negative) of the **2**–malonate complex. Inset: calculated isotopic pattern for $C_{25}H_{18}BO_6^-$.

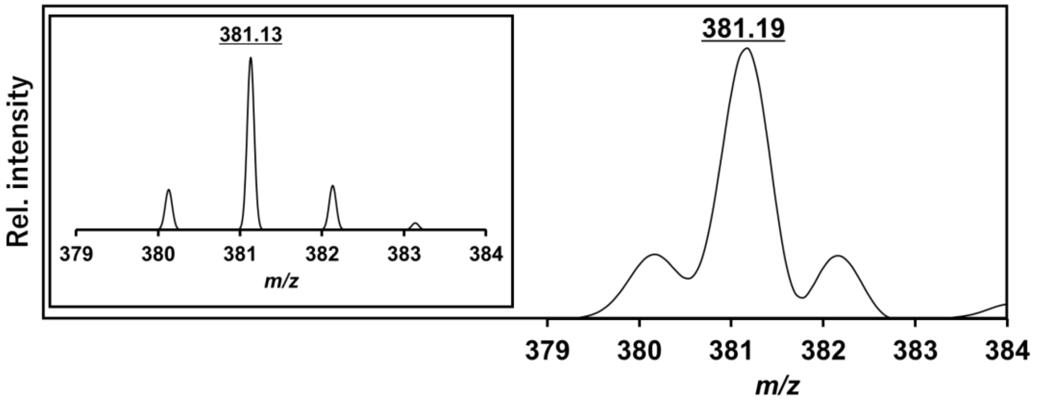

Supplementary Figure 25. ESI-MS spectrum (negative) of the **2**–acetate complex. Inset: calculated isotopic pattern for $C_{24}H_{18}BO_4^-$.

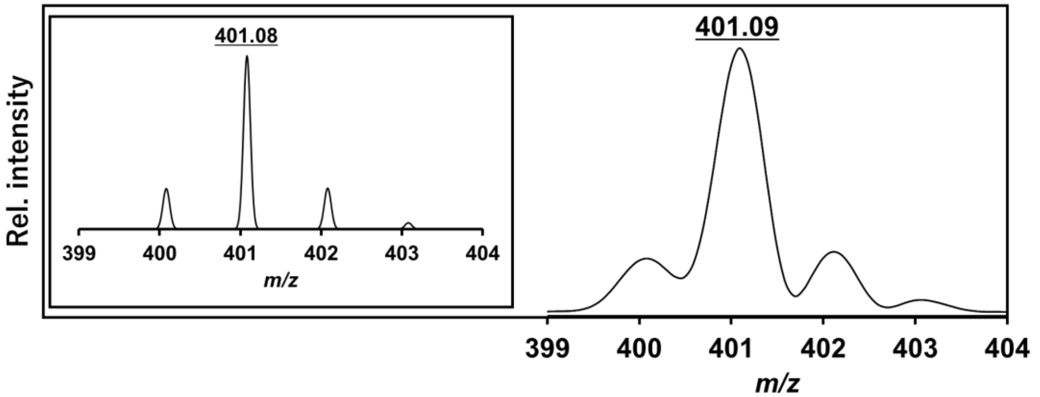

Supplementary Figure 26. ESI-MS spectrum (negative) of the **2**–Pi complex. Inset: calculated isotopic pattern for $C_{22}H_{15}BO_5P^-$.

3. Linear Discriminant Analysis (LDA)

Supplementary Table 1 Jackknifed classification matrix of the qualitative assay.

	Acetate	CA	F^-	Pi	Mal	Oxa	Control	PPi	%correct
**Acetate**	20	0	0	0	0	0	0	0	100
**CA**	0	20	0	0	0	0	0	0	100
**F^-**	0	0	20	0	0	0	0	0	100
**Pi**	0	0	0	20	0	0	0	0	100
**Mal**	0	0	0	0	20	0	0	0	100
**Oxa**	0	0	0	0	0	20	0	0	100
**Control**	0	0	0	0	0	0	20	0	100
**PPi**	0	0	0	0	0	0	0	20	100
**Total**	20	20	20	20	20	20	20	20	100

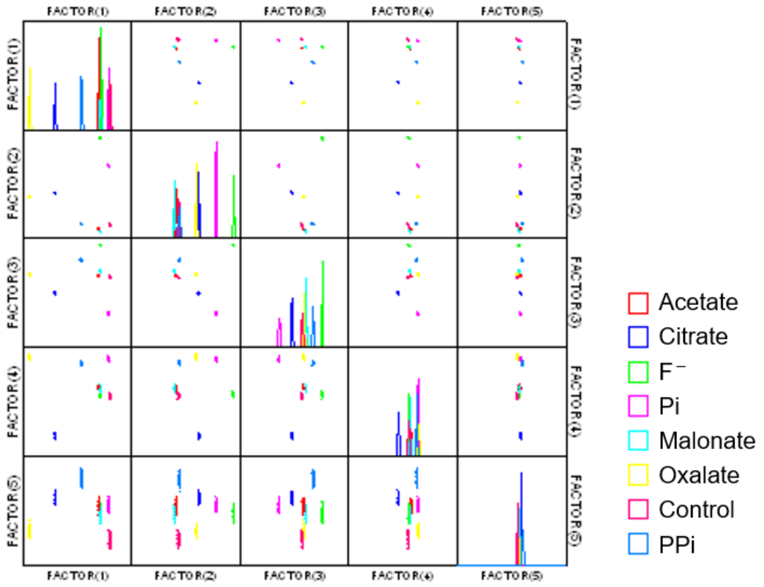

Supplementary Figure 27. The canonical score plot of quantitative assay.

Supplementary Table 2 The jackknifed classification matrix of semi-quantitative assay.

	CA-0.01 mM	CA - 0.02 mM	CA-0.04 mM	CA-0.07 mM	CA-0.10 mM	Mal-0.01 mM	Mal-0.03 mM	Mal-0.05 mM	Mal - 0.10 mM	Oxa-0.01 mM	Oxa-0.02 mM	Oxa-0.03 mM	Oxa-0.05 mM	Oxa-0.06 mM	Oxa-0.07 mM	Oxa-0.09 mM	Oxa-0.10 mM	Ctrl	% Correct
**CA-0.01 mM**	20	0	0	0	0	0	0	0	0	0	0	0	0	0	0	0	0	0	100
**CA - 0.02 mM**	0	20	0	0	0	0	0	0	0	0	0	0	0	0	0	0	0	0	100
**CA - 0.04 mM**	0	0	20	0	0	0	0	0	0	0	0	0	0	0	0	0	0	0	100
**CA - 0.07 mM**	0	0	0	20	0	0	0	0	0	0	0	0	0	0	0	0	0	0	100
**CA - 0.10 mM**	0	0	0	0	20	0	0	0	0	0	0	0	0	0	0	0	0	0	100
**Mal-0.01 mM**	0	0	0	0	0	20	0	0	0	0	0	0	0	0	0	0	0	0	100
**Mal-0.03 mM**	0	0	0	0	0	0	20	0	0	0	0	0	0	0	0	0	0	0	100
**Mal-0.05 mM**	0	0	0	0	0	0	0	20	0	0	0	0	0	0	0	0	0	0	100
**Mal-0.10 mM**	0	0	0	0	0	0	0	0	20	0	0	0	0	0	0	0	0	0	100
**Oxa-0.01 mM**	0	0	0	0	0	0	0	0	0	20	0	0	0	0	0	0	0	0	100

**Oxa-0.02 mM**	0	0	0	0	0	0	0	0	0	0	20	0	0	0	0	0	0	0	100
**Oxa-0.03 mM**	0	0	0	0	0	0	0	0	0	0	0	20	0	0	0	0	0	0	100
**Oxa-0.05 mM**	0	0	0	0	0	0	0	0	0	0	0	0	20	0	0	0	0	0	100
**Oxa-0.06 mM**	0	0	0	0	0	0	0	0	0	0	0	0	0	20	0	0	0	0	100
**Oxa-0.07 mM**	0	0	0	0	0	0	0	0	0	0	0	0	0	0	20	0	0	0	100
**Oxa-0.09 mM**	0	0	0	0	0	0	0	0	0	0	0	0	0	0	0	20	0	0	100
**Oxa-0.10 mM**	0	0	0	0	0	0	0	0	0	0	0	0	0	0	0	0	20	0	100
**Ctrl**	0	0	0	0	0	0	0	0	0	0	0	0	0	0	0	0	0	20	100
**Total**	20	20	20	20	20	20	20	20	20	20	20	20	20	20	20	20	20	20	100

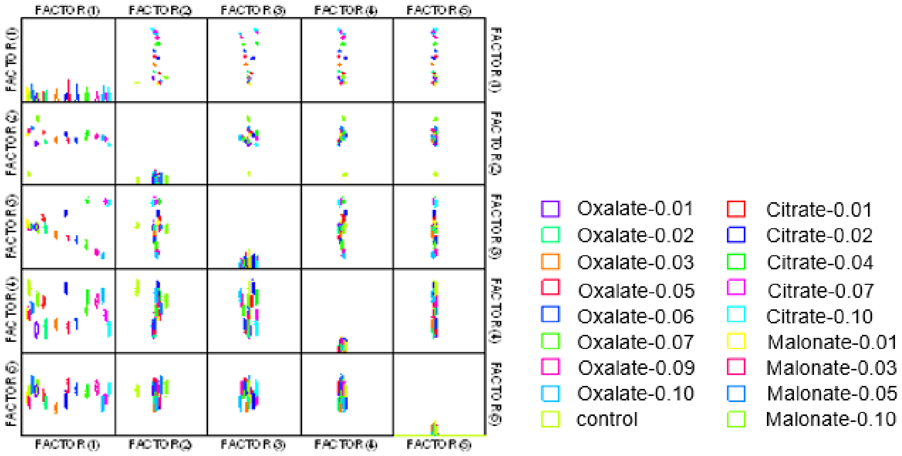

Supplementary Figure 28. The canonical score plot of semi-quantitative assay.

Supplementary Table 3 Concentration conditions in the quantitative assay of the mixtures. The gray and white lines mean validation and calibration data sets, respectively.

Malonate (mM)	Citrate (mM)	Oxalate (mM)
0.01	0.01	0.01
0.02	0.02	0.02
0.03	0.03	0.03
0.04	0.04	0.04
0.05	0.05	0.05
0.06	0.06	0.06
0.07	0.07	0.07
0.08	0.08	0.08
0.09	0.09	0.09
0.1	0.1	0.1
